# Supplementary material for: Autism, thy name is man: Exploring implicit and explicit gender bias in autism perceptions
Source: PLoS One. 2023 Aug 23;18(8):e0284013. doi: 10.1371/journal.pone.0284013 (PMC10446214; doi:10.1371/journal.pone.0284013)
Supplement: Attachment — Submitted filename: Manuscript_1+reviewer.docx [file pone.0284013.s001.docx]

Title: Autism, thy name is man: Exploring implicit and explicit gender bias in autism perceptions

Short title: GENDER BIAS AUTISM 111

Rae Brickhill* , Gray Atherton , and Liam Cross

1 Edge Hill University

*Corresponding author information: Rae Brickhill, St Helens Rd, Ormskirk L39 4QP, UK, (e-mail: rae.brickhill@btinternet.com).

Abstract:

Males are an estimated three times more likely to possess an autism diagnosis than females. For years this was explained by accounts that considered the male gender more compatible with the autistic phenotype. However, new research suggests that a lack of understanding and recognition of the female autistic phenotype, and a predisposition to associate males rather than females with autistic traits, could lead to structural inequalities that hinder the identification of autistic females. To explore how autism and gender are more widely perceived, the present study tested implicit and explicit associations between autism and male and female identities using the implicit association test (IAT) and the AQ when presented alongside a male or female vignette. A significant association was found on the IAT identifying an implicit bias towards males and autistic traits. The AQ also revealed specific items that were perceived as explicitly male traits, while only reverse scored items were perceived as female. These findings suggest that current perceptions and even metrics of autism are skewed towards males rather than females, which may in and of itself hinder the identification and understanding of the female autistic phenotype.

Keywords:

autism, gender, implicit bias, attitudes, Autism Quotient, implicit association task, autism quotient

Data availability statement:
The data that support the findings of this study are available on request from the corresponding author. The data are not publicly available due to privacy or ethical restrictions.

Autism Spectrum Condition (ASC) is a complex neurodevelopmental condition characterized by impairments in communication and social interaction (American Psychiatric Association, 2013). While this neurodevelopmental disorder has been recognized by Kanner (1943) since the 1940s, autism research has seen drastic growth in the past several decades, and is continually on the rise due to autism’s increasing prevalence (Ruzich et al., 2015). In line with other neurodevelopmental conditions, autism is more commonly diagnosed in males than females (Park et al., 2016), with three times more males diagnosed than females (Loomes et al., 2017). Females are frequently diagnosed later in life than their male peers (Giarelli et al., 2010) and have an increased risk of remaining undiagnosed (Bargiela et al., 2016; Lai et al., 2015). This has led some to question whether autism is perceived as being a ‘male’ condition at its most basic level and thus leads to systematic discrimination against females who are autistic. This is a point that has been raised by those in the autistic community, such as parents of autistic female children (Milner et al., 2019), women who received a diagnosis later in life (Bargiela et al., 2016), and autistic adults with experiences of gender dysphoria (Coleman-Smith et al., 2020).

**Gender stereotypes**

Research suggests that behaviours and perceptions of men and women are governed by stereotypes, as they provide prompt assumptions toward members of social groups (Amodio, 2014). For example, males are perceived to have more agentic characteristics than females, as they are perceived to be more independent, assertive, and dominant; while increased empathy and greater communality are perceived in females (Garcia-Retamero et al., 2009; Hess et al., 2000; Kite et al. 2008). Females have also been found to be more emotionally expressive than men while being better able to demonstrate greater use of non-verbal communication both in-person and during online interactions (Briton & Hall, 1995; Parkins, 2012). Shyness is also a common trait associated with females; for instance, Geelhand et al. (2019) found shyness to be much less likely to raise concerns for girls in school than boys due to the social expectation that girls are shy. These expectations can then influence individual’s perceptions of men and women and how they act (Eagly & Wood 2013; Ellemers, 2018). Jackson and Bussey (2020) corroborate this. They compared the pressure felt by adolescents to engage in same-gender behavior; extending the current understanding of gender identity, as men reported pressure to avoid feminine-typed behavior. On average, females are also believed to be more susceptible to social pressures than males (Pascual-Miguel et al., 2015).

**Gender stereotypes and autism**

Female stereotyped behaviors in many ways stand in stark contrast to typically recognized autistic traits. Overall, the language used to describe ASC is littered with certain gender expectations. For instance, the general view that women display more heightened emotional expressions alongside greater motivation to communicate for social reasons than men directly contrasts with characteristics associated with autism (Krahn & Fenton, 2012). Such biases may contribute to the systematic constraints that prevent girls from obtaining recognition for their ASC from their parents, educators, and peers at the same rate as their male counterparts. A reluctance to view a female as autistic likely has a detrimental effect on wellbeing. Improved understanding and more positive perceptions from peers is an essential outcome of a recognized diagnosis (Matthews et al., 2015).

Recognising an individual as autistic is critical for improving double empathy between the autistic person and the neurotypical community (Milton, 2012), as it allows neurotypicals to recognize that an autistic person possesses a neurodivergent perspective that is equally deserving of understanding and respect. Failure to acknowledge and validate neurodivergent views is referred to as the double empathy problem, and it may be compounded in autistic females. Specifically, as the stereotypes associated with females may be particularly at odds with perceptions of autism, there runs the risk of a heightened misunderstanding of neurodivergence in females. For instance, qualitative research from Bargiela et al. (2016) revealed the ways in which healthcare providers consistently dismissed autistic female clients’ accounts of their autistic traits as being more indicative of conditions such as depression with higher female ratios, while autistic participants in Coleman-Smith et al. (2020) discussed the ways that their experiences of gender dysphoria were minimized or explained away by their autistic status. These accounts show how stereotypes of neurodivergence and gender can both reduce double empathy and inhibit acceptance of autistic people who may not fit the typical male-autistic presentation.

There are several dominating accounts of the social features of autism that are at odds with female stereotypes. The theory of mind account (Baron-Cohen et al., 1985), which cites autistic social differences as stemming from poor perspective taking, may also reflect a gendered stereotype that favours males. Females are believed to be more likely to take others perspective into account (Heilman & Chen, 2005), and indeed ToM tasks often reveal a female advantage using both behavioral (Devine & Hughes, 2013) and neural methods of investigation (Adenzato et al., 2017). Another popular theoretical account of the social features of autism, the social motivation account (Chevallier, 2012) purports that autistic people are less interested in social stimuli than is typical. Similarly, research suggests that females are increasingly socially motivated than males (De Goede et al., 2009; Luengo Kanacri et al., 2013).

Interestingly, research that uses both male and female autistic samples have not found a female advantage on social tasks. For instance, autistic females appear to have similar difficulties reading emotions in eyes as autistic males, despite an advantage on the same task in neurotypical females compared to males (Baron-Cohen et al., 2015). In an effort to minimize differences in ways that match society’s expectations, autistic individuals often camouflage their traits (Attewood, 2007) by attempting to emulate the social interaction style of neurotypical individuals (Hull et al., 2020) to fit in more with their peers (Kenyon, 2014). Motivation for camouflaging and the masking of social difficulties is increased by social pressures and expectations experienced by females (Hull et al., 2017). For instance, women with autism ‘pretend to be normal’, by copying others’ speech patterns and body language (Bargiela et al., 2016; Willey, 2014) as a reaction to the societal messages they receive, which tell them as female they should be more sociable than males (Milner et al., 2019). There is a worrying association between camouflaging, ASC traits, and diminished mental health (Cassidy et al., 2020), alongside the stress associated with autism being ‘found out’ (Cage & Troxell-Whitman, 2019). These additional gendered pressures are of pragmatic concern. One way to improve these outcomes for women on the spectrum is to understand and recognize autism in females, particularly as it concerns early diagnosis. Equal identification of female autistic traits is imperative as, among other benefits, those who are recognized as autistic experience greater acceptance (Matthews et al., 2015) as there is a reason and explanation for expressing atypical behavior (Matthews et al., 2015).

Understanding discrepancies in male and female identification rates is particularly imperative in the early years, as research suggests early intervention has many benefits, including greater gains in communication and wellbeing (Mazurek et al., 2019). Unfortunately, there are systematic male biases that negatively impact the early identification of autistic female children. For instance, Dean et al. (2016) found gender-related assumptions about the level of social engagement and activities of male and female children with and without ASC at school. Specifically, the social challenges of boys with ASC were viewed as more obvious than those of girls with ASC. This correlates with research conducted by Whitlock et al. (2020), who found that educators within a school setting expressed reduced likelihood in seeking support for a female believed to have autism compared to an equivalent male. Finally, parents’ perception of their child’s behaviours and difficulties may also affect an individual’s motivation to camouflage, due to social expectations varying depending on the child’s gender (Young et al., 2018). This poses serious consequences for the wellbeing of females with ASD, putting them at an unjust disadvantage to males in educational and domestic contexts (Giarelli et al., 2010).

The consequence of gendered stereotypes’ being at odds with typical characteristics of autism is that they can then fuel common misconceptions of autism within the general population. Such stereotypes can result in unfavorable inferences being made about individuals with ASC based on their social, behavioral, and communicative differences, which can be detrimental to their wellbeing (Cage et al., 2018). For instance, people form a more negative first impression of individuals with ASC than those without, whilst also being influenced by the individual’s gender, with large differences apparent between the judgements of autistic females compared to non-autistic females (Cage & Burton, 2019). This bias has also been found to occur at an unconscious, implicit level through the use of an Implicit Association Test (IAT) (Greenwald et al., 1998), in which individuals are timed on their ability to pair a set of stimulus words with a similar meaning (i.e. ‘good,’ ‘nice,’ ‘kind’) with a categorical word (i.e. ‘Muslim’ ‘British’). In an IAT test looking to test the association between autism and positive and negatively valenced words, Dickter et al. (2020) identified significant negative implicit attitudes towards autistic adults in the general population, despite participants displaying positive explicit attitudes. These results provide further explanation for the discrimination against autistic adults within society. Given the stereotypical associations between female traits and female sociality, discrimination may be particularly heightened towards autistic females.

Gendered stereotypes towards autism are also reinforced within the media as stereotypes and stigmas surrounding autism can be strengthened (Nordahl-Hansen et al., 2018), typically through the use of male characters as seen in *The Curious Incident of the Dog in the Night-Time,* *Rain Man, The Big Bang Theory* and *Atypical*. These popular representations can influence understanding stereotyped neurodevelopmental disorders, such as autism, even when an individual has personal experience of the condition (Garner et al., 2015). This is important to consider when studying the general population as they may not have access to personal experience, suggesting further reliance on the media to understand autism, further contributing to stereotyped attitudes. Furthermore, Garner et al. (2015) report the representation of only “very high levels” to “extreme levels” of autism-related symptoms within the media. This may further contribute to the high levels of missed diagnosis of females with less immediately apparent areas of functional need. Thus, the general public may form a highly stereotypical view of individuals with ASC, often through the lens of male rather than female characters.

**Gender biases in autism research**

Gender bias has likely filtered into academic research, resulting in a lack of sensitivity towards the symptomology presented by ASC females (Lai et al., 2015), as the current diagnostic tools for autism are believed to be geared towards the male autism phenotype (Kopp & Gillberg, 2011). Some of this may stem from the fact that autism research favours male over female participants (Lai et al., 2015). This sampling bias is a likely contributor to why male presentations of autism are more often used as a guide for the design and implementation of current diagnostic tools, which exacerbates the misidentification of autistic females (Mandy & Lai, 2017). These challenges may explain why females with ASC must present with a higher level of functional needs (such as more concurrent behavioral, developmental, or mental health issues) in order to be diagnosed compared to males (Duvekot et al., 2017; Lehnhardt et al., 2015). Indeed, among studies with autistic individuals without intellectual disability, the ratio between males to females is as high as 10:1 (Dworzynski et al., 2012).

Some suggest that there is one specific theory of autism that perpetuates male autism stereotypes and lacks sensitivity towards the female autism phenotype (Yaull-Smith, 2008). The ‘extreme male brain’ theory of autism suggests that, due to their systemising nature and hindered emotional responses (Barron-Cohen., 2002), autistic people are an example of a hyper-masculinized group of individuals regardless of gender. Although Greenberg et al. (2018) emphasise that the term ‘extreme male brain’ simply predicts that individuals with autism will show a masculinized pattern of scores of reduced empathy and above average systemizing rather than possessing an extreme of all masculine characteristics (such as aggression), the gendered term may affect how the general population views autism and how they associate the disorder with males.

Ridley (2019) argues that within the term ‘extreme male brain’, the use of ‘brain’ has replaced ‘behaviour’, with no explanatory power, causing confusion and little descriptive utility. Further questioning the use of this term, Ruzich et al. (2015) compared the AQ scores of neurotypical males and females with the scores of autistic males and females - finding that ASC females’ scores were only similar to the scores of ASC men and not those displayed by neurotypical men. This suggests that the scores produced by both males and females with ASC are the result of the neurodevelopmental condition itself, rather than possessing a ‘male brain’, as neurotypical men did not produce similar scores to either males or females with ASC. Unsurprisingly, the misinterpretation of this term can have profound impacts on societal perceptions of autism in males and females, by encouraging a gendered bias and understanding towards the disorder. Indeed, qualitative accounts of autistic people with gender dysphoria have specifically mentioned the way that such accounts exacerbate their feelings of identity confusion (Coleman-Smith et al., 2020).

While it is assumed that individuals carry a gendered view of autism due to the proliferation of male media portrayals of autistic people, and gendered stereotypes of females that are often in direct contrast to autistic characteristics and popularized theoretical accounts of autism as a masculinized condition, few have directly tested this relation. It is essential to assess the views of the general population, as although attitudes towards autism may be improving (White et al., 2019), stigma remains common (Gillespie-Lynch et al., 2020) and individuals’ biases towards autism can often contribute to personal and professional challenges for autistic people (Jones et al., 2021). This population-based study explores whether gender biases the perception of autism on implicit and explicit measures.

**The Current Study:**

In order to measure how gender colours the perception of autism at the subconscious level, an implicit association test (IAT) (Greenwald et al., 1998) was employed to measure participants’ implicit gendered autism associations by recording their reaction times when categorizing male and female names with autistic traits to observe potential implicit bias between the two categories. It was hypothesized that the association between autistic characteristics will be stronger with males than females in an IAT. Autism Spectrum Quotient (AQ) scores in relation to a male or female vignette were compared through a MANOVA to explore whether certain traits on the AQ will be more heavily endorsed when the AQ is being answered about a hypothetical male than a female.

**Methods**

## **Design & Participants**

This study employed a mixed design comparing Neurotypical/ASC - female/male implicit associations within participants and explicit endorsement of fe/male targets on an AQ between participants. A power analysis using G*Power determined that 300 participants (*n* = 300) would be needed to detect a small effect size of Cohen’s *d* = 0.3. This sample size was preregistered prior to data collection, along with hypothesis and analysis plans (<https://aspredicted.org/blind.php?x=8xn4v6>). Participants (n = 300, 149 male, 151 female, age range 18 to 72 years, *M* = 26.79 *SD* = 10.113, 81.27% White, 2.22% Black or African American, 12.70% Asian, 3.81% Other) were recruited from online research and social media platforms. They were required to be over the age of 18 to partake in the study. All participants gave full informed consent, were above the age of 18, and the XXX-BLINDED FOR REVIEW-XXXethical review board granted ethical approval. Of the 300 participants, 14 individuals possessed a medical diagnosis of autism (of which 8 completed female condition and 6 completed the male condition).

**Materials & Procedure**

Implicit associations were assessed using an IAT employed on the study platform Qualtrics (Provo, UT). Specifically, participants were presented with target (male / female name) and attribute (autistic and non-autistic traits) words, one at a time in the centre of the screen, instructed to press ‘E’ on the keyboard when the picture or word matched the target variable on the left, and ‘I’ when the term matched the target variable on the right. On crucial trials, male/autistic and female/non-autistic words were categorized using the same key. On the remaining trials, the pairings of attributes and targets were switched. Incorrect responses presented participants with a black screen and a red ‘X’, and they were then prompted to press the correct key. The speed at which participants accurately categorized the words and the number of errors was recorded. Reaction time and accuracy in response to each attribute pairing showed a stronger implicit association between the two items.

A 7-block IAT was used, with each block consisting of 20 trials (Nosek et al., 2002), and in line with recommendations, all trials with reaction times under 300 ms were removed (Lane et al., 2007). Errors made within each trial were replaced with a participant’s mean reaction time for that block, plus an error penalty of 600 ms (Lane et al., 2007). Participants' mean reaction time for each association pairing, male autistic/female non-autistic and female autistic/male non-autistic were then calculated. This produced a total reaction time of both the female autism pairing and male autism pairing, which accounted for both reaction times and errors.

IAT stimuli consisted of eight autistic and eight neurotypical (non-ASC) traits and eight male and eight female names. The names used within the IAT were chosen from amongst the top 100 baby names in the 2000s due to the projected age of the majority of participants completing the study (BabyNames.it, 2021). Names were also chosen based on their similarity and shortness; short items within an IAT are favored in order to reduce error and increase participant response times (Greenwald et al., 2009). The ASC/NT items were chosen based on previous research by Obeid et al. (2020) which used a similar IAT to investigate implicit racial biases and how they influenced autism recognition. In keeping with the recommendations that IAT stimuli be short, some of the original items were reduced in word count. For example, ‘displays rigid routines’ was shortened to simply ‘rigid.’ Further IAT and autism research conducted by Lipson et al. (2020) provided additional items for the autism category such as ‘introverted’, ‘autistic’, ‘atypical’ and ‘different’, whilst neurotypical items were identified as ‘extraverted’, ‘flexible’, ‘typical’, ‘independent’ and ‘social’. Pilot research conducted by Lipson et al. (2020) provides support for these choices of words, as students were asked what behaviours they think are characteristic of an autistic individual. Broader research on autism also suggests that autistic individuals have difficulties with social reciprocity and expressing emotion (APA, 2000; Losh & Capps, 2006); supporting the ‘rigid,’ ‘introverted,’ ‘reserved,’ and ‘different’ items. IATs require closely related items to comprise the two stimulus categories, to make it easier for participants to respond differentially (Kim, 2003). Therefore antonyms of the autistic word items were used for the words in the non-autistic category, such as introverted (autistic) and extraverted (non-autistic). A complete list of all items used can be found in Table 1.

Table 1. *Representation of IAT categories*

| Category | Items |
| --- | --- |
| Male | Josh, Jack, Tom, James, Dan, Ben, Luke, Ryan |
| Female | Emma, Chloe, Sarah, Kate, Amy, Lisa, Emily, Zoe |
| Autistic | Autistic, Reserved, Rigid, Asperger's, Atypical, Different, Logical, Introverted |
| Non-Autistic | Neurotypical, Spontaneous, Flexible, Typical, Extraverted, Social, Talkative, Outgoing |

In addition to the IAT, the 50-item AQ (Baron-Cohen et al., 2001) along with an accompanying vignette was used to measure explicit associations between autism and male and female genders. The AQ is a screening tool devised to measure the degree of autistic traits an individual may possess. The AQ provides participants with 50 statements, which they are required to rate in terms of how much they agree or disagree with the statement in relation to a 4-point Likert-type scale ranging from 1 (strongly disagree) to 4 (strongly agree).

The AQ scores range from 0 to 50, with a higher score relating to a higher degree of autistic traits through both social (“I prefer to do things with others rather than on my own”) and non-social (“I am fascinated by dates”) aspects of cognition and behaviour (Ruzich et al., 2015, and half of the items are reverse scored. The present study implemented the AQ to assess how participants perceived either a male or female individual described in a preceding vignette. Images were also provided alongside both vignettes, showing either a male or female face. These pictures were obtained from the Karolinska Directed Emotional Faces (KDEF) (Lundqvist et al., 1998) to further prime the gender of the character in the vignette.

The vignettes described an individual with autistic traits and were designed based on overarching autistic characteristics identified in previous research (Whitlock et al., 2020). Specifically, Whitlock et al. (2020) developed vignettes through an iterative process of consulting autistic adults, clinicians, researchers and educators. The outcome of this research provided the following autistic traits: difficulty socialising, restrictive interests and difficulty with change. Immediately after completing the IAT, participants read the following vignette half (in the male condition/half female), after which they respond to the AQ-50 statements, as if they were the individual described in that vignette.

“*Matthew/Kerry* is very reserved. *He/she* struggles to make new friends as *he/she* prefers to speak to people *he/she* is familiar with and prefers to speak to people *he/she* already knows. *He/she* doesn’t like it when *his/her* desk is rearranged at work and likes having a routine. At work, *Matthew/Kerry* has difficulty determining if *his/her* boss is happy with *his/her* performance and he/she often struggles to understand *his/her* colleague's jokes. *He/she* prefers speaking about topics he/she knows a lot about. In *his/her* leisure time, *Matthew/Kerry* plays individual games and sports, like golf, rather than playing games in teams.”

# **Results**

|  | Mean Response Times |
| --- | --- |
| Female | 2161.73 |
| Male | 2015.74 |

Mean and standard errors of the mean reaction times for the male and female category

A pre-registered paired samples *t*-test was used to explore the mean RT for each set of association pairings. Response times were significantly lower when autistic traits were being attributed to males (*M* = 2015.7406, *SD* = 524.78081) compared to females (*M* = 2161.7320, *SD* = 803.96293), *t*(299) = -3.540, *p* < .001; *d* = -0.2197564, indicating that participants found it easier to associate autistic words with male names. This relative ease suggests that individuals have an implicit bias towards males and autistic traits. Participants were better at categorising autistic traits with male names (suggesting stronger associations in this direction). These empirical findings reveal a tendency in the average person to associate autistic traits with males compared to females.

Exploratory research was conducted to ascertain whether implicit associations were related to the level of autism knowledge. A Pearson’s correlation coefficient measured a possible correlation between participants' knowledge of autism and their mean reaction times. No correlation was found for male mean reaction times *r* = 0.057, *n* = 127, *p* = .527, or for female mean reaction times *r* = 0.028, *n* = 299, *p* = .631, suggesting knowledge of ASC was not a protective factor against a male autism bias.

A preregistered between-subjects MANOVA was then carried out, testing the main effect of gender (male or female condition) on the level of perceived autistic traits assigned to the individual described in the vignette. One AQ item was identified as differing significantly across the conditions at the .05 alpha level, and a further 3 at the .01 alpha level. Bayesian T-tests were then conducted on marginally significant items to evaluate the likelihood of H1 over H0. BF01 evaluates the Research hypothesis while BF10 the Null hypothesis. See Table 2 for descriptive and inferential. All other items were found to be comparable across conditions (p’s >.01).

|  | **Mean** | | | | ***SD*** | | | ***f* Value** | ***p* Value** | **Partial Eta Squared** | | **BF_1_** | | **BF_10_** |  |
| --- | --- | --- | --- | --- | --- | --- | --- | --- | --- | --- | --- | --- | --- | --- | --- |
|  | **Male** | **Female** | | **Male** | | **Female** | |  |  |  | |  | |  |  |
| 1. I prefer to do things with others rather than on my own. | 2.67 | | 2.99 | 1.22 | | 1.20 | 5.238 | | .023 | .017 | | - | | - |  |
| 34. I enjoy doing things spontaneously | 3.36 | | 3.52 | 0.82 | | 0.75 | 3.014 | | .084 | .010 | | 7.356 | | 0.136 |  |
| 39. People often tell me that I keep going on and on about the same thing. | 3.24 | | 3.06 | 0.84 | | 0.78 | 3.709 | | .055 | .012 | | 1.348 | | 0.742 |  |
| 41. I like to collect information about categories of things (e.g., types of birds, cars, trains, plants). | 3.24 | | 3.08 | 0.85 | | 0.82 | 2.769 | | .097 .009 | |  | | 2.105 0.475 | | |

Table 2. *MANOVA results of significant of AQ-50 items*

# **Discussion**

The present study investigated whether those in a general population sample displayed a male gender bias in their perceptions of autism. A significant implicit gender bias towards associating males with autistic traits was indeed found on using both implicit and explicit measures. This implicit association is consistent with a higher male-to-female autism ratio and evidence that autistic females are at a greater risk of remaining undiagnosed (Bargiela et al., 2016; Loomes et al., 2017).

This implicit bias towards males and autistic traits may have a detrimental effect on the diagnosis rate of females and have profound effects on their wellbeing (Giarelli et al., 2010) and exacerbate masking, making ASC females less likely to receive clinical support (Dworzynski et al., 2012). The development of sexual identity may also be affected by the heavily gendered view of autism, as research identifies a key emerging theme of gender dysphoria and a sense of conflict surrounding sexual identity within autistic individuals (Coleman‐Smith et al., 2020). For instance, in previous research, gender dysphoric autistic participants have expressed how ‘gender-loaded stereotypes’ surrounding autism complicated their understanding of their own gender (Coleman‐Smith et al., 2020).

The extent of this issue is further highlighted in qualitative research conducted by Bargiela et al. (2016) and Milner et al. (2019). Both reports identify females with ASC experiencing feelings of ‘not fitting in’ with society, as their atypical behaviors did not comply with their peers and remained unaccounted for by a lack of a diagnosis of autism. The concept of gender was also frequently discussed in both studies with specific mention of the pressure placed on females ‘to be a certain way’ – highlighting the detrimental effects of gender bias on autistic female wellbeing. For instance, being quiet and passive within a group was seen as more socially acceptable for females compared to males, leading to their symptoms’ remaining hidden (Bargiela et al., 2016). Overall, the suggestion that males with ASC experience less pressure to camouflage their symptoms were critical features of both studies. Indeed, females with ASC recognize this as a contributing factor to the difference in diagnosis rates between males and females (Milner et al., 2019).

This study revealed that the language used to describe ASC may be incongruent with certain gender expectations for females. As revealed in both the IAT and the AQ-50, the general view that women pose more emotional traits alongside greater motivation to communicate for social reasons than men appears to stand in direct contrast to features associated with autism (Krahn & Fenton, 2012). The current study provides quantitative support for this, by revealing gender biases towards males and autism, as it identifies highly significant difference in people’s implicit bias towards males and ASC compared to females, alongside evidence of explicit views towards females with ASC being perceived as more social than males with ASC.

Using a widely used early screener for adults seeking an autism diagnosis, the AQ-50, an explicit bias was demonstrated in the current study, supporting the notion that certain items on the measure are more indicative of male versus female traits. Results from Question 1 on the AQ-50 (“I prefer to do things with others rather than on my own”) indicate that females were perceived as more likely to be interdependent than men whilst question 34 (I enjoy doing things spontaneously) also highlights that females are perceived as more spontaneous than males. Indeed, no autistic items in the AQ-50 were rated more highly for the female vignette than the male vignette. This suggests an explanation for why girls and women often need to present with more concurrent behavioural, developmental or mental health issues for an autism diagnosis would be made, compared to their male counterparts in the current diagnostic practice (Duvekot et al., 2017; Dworzynski et al., 2012); thus contributing to females with ASC remaining undiagnosed (Loomes et al., 2017). It also suggests that commonly used self-report measures are not representative of female autistic traits that may be differentiable from male autistic traits (Kirkovski et al., 2013).
 Similarly, question 39 (people often tell me that I keep going on and on about the same thing) was rated higher for males. This is consistent with the gender bias that females are perceived to have greater overall social skills and are more able to intuit social cues more adeptly than men. This may explain females’ increased ability to camouflage their ASC traits (Rynkiewicz et al., 2016; Wood-Downie et al., 2020) and their tendency to display more socially acceptable restricted interests (Frazier et al., 2014). Camouflaging is also believed to be enhanced by the increased social pressures and expectations that women face compared to males in everyday life (Bargiela et al., 2016; Geelhand et al., 2019; Milner et al., 2019), as females mask their traits in order to fit in more within society and gain a greater understanding and acceptance from their peers due to a dissociation between their gender and diagnosis.

Increased motivation to camouflage due to societal pressures (Kenyon, 2014) can therefore contribute to the later medical diagnosis of autism received by females compared to males, as clinicals may be implicitly influenced by gendered views towards autism and males; thus, putting ASC females at increased risk of developing anxiety and depression (Giarelli et al., 2010; Pellicano et al., 2014). This poses severe consequences for the wellbeing of females with ASC, putting them at a disadvantage to males (Giarelli et al., 2010). Gray (2002) states that females with ASC are often viewed as more responsible for their atypical behaviours, leading to a greater suffering of anxiety, depression, and lower self-esteem in comparison with autistic males (Watson, 2014).

Additionally, question 41 (I like to collect information about categories of things e.g., types of cars, birds, trains, plants) was rated more highly for the male vignettes. This perception may be due to the categories suggested by the question itself (such as cars and trains). These items are more likely to represent male interests which may automatically lead to more male-dominated assumptions about ASC. For instance, girls with ASC have been found to present with different or more socially appropriate restricted interests than boys (Attwood et al., 2006), such as an obsession with a specific television character/celebrity, whereas boys’ interests revolved around wheeled toys (Hiller et al., 2014). Similarly, Sutherland et al. (2017) found autistic boys to have interests that fit more traditional gender stereotypes than autistic girls. This suggests that the restricted interests presented in an individual with ASC are also affected by societal expectations and bias. The restricted interests found in boys and girls in research conducted by Hiller et al. (2014) followed interests that are arguably typical with their gender. These results, therefore, suggest that socially constructed gender biases shape how ASC related behaviours are perceived (Lai et al., 2015). This highlights the importance of changing attitudes towards females presenting with autism to create wider acceptance and acknowledgment and reduce the need to camouflage and mask autistic traits and potential gender identity issues. This would improve the social and developmental outcomes for females with ASC, while reducing their likelihood of developing mental health issues.

There were limitations to this study that could be addressed by further research. First, this study was conducted with an online sample of individuals from the general population. While it was interesting to understand how the general public genders autistic individuals regarding community perceptions and acceptance of autistic people, there are other groups for whom a gendered bias towards autistic males rather than females would be particularly detrimental. Replicating this study with autism professionals is a necessary extension of this work. Understanding male versus female biases in healthcare providers, educators, and clinicians is of specific interest. Such individuals are often the first to identify individuals who may qualify for a diagnosis. It would also be interesting to investigate gender biases among autism researchers, an important consideration given that autism studies have a history of recruiting predominantly male samples. As autism researchers are responsible for creating the measures and interventions widely used to assist autistic individuals, understanding whether there are systematic biases that color such tests and programs is an important next step in improving upon these shortcomings.

Additionally, this study used a vignette that was interchangeably presented to represent both a hypothetical male and female. While vignettes are often used in research to understand social biases by describing overt behaviors, they cannot present the more subtle behaviors often associated with ASC, including verbal and non-verbal communication differences. Video recordings of autistic males and females used to uncover gender biases towards the perception and acceptance of autistic males and females would be appropriate in future. As our research suggests that autistic traits conflict with commonly held stereotypes regarding female versus male social traits, it may be that more sensitive stimuli lead to greater discrimination against autistic females, which would help explain higher prevalence of masking. Second, while the same vignette was used to create a clear comparison between male and female conditions, it could be argued that it was more representative of male autistic traits, as this study suggests that what is understood to be an ‘autistic’ trait is itself male-biased. Future research may want to replicate this work with vignettes that showcase more typically female autistic traits (i.e. restricted interests that are more commonly female, such as animals or celebrities, and social behaviors such as shyness). Such a vignette may reveal even more pronounced discrepancies between males and females regarding popular measures such as the AQ, leading to restructuring existing standards or creating more balanced tests.

In conclusion, the results of this study highlight the effects of gender bias and gender-based expectations on the perceptions of ASC. This adds to the recent subdiscipline of autism research aimed at elucidating the nature of sex and gender differences on the autism spectrum, and how this impacts autistic girls and women (Lai et al., 2015). The current research provides further evidence that the general public holds gendered views of autism and that typical measures of autistic traits are biased towards males over females. Understanding how this bias extends to autism professionals who are particularly critical in identifying and supporting autistic individuals is paramount, as revealing such a bias could help explain the discrepancies between autistic male and female ratios. Research also suggests that autistic people are significantly more likely to experience gender dysphoric perceptions compared to neurotypicals. As gendered roles are learned through interaction with societal norms, and this research suggests that society views autism as a male rather than a female manifestation, it would be interesting to examine how this contributes to gender dysphoria in autistic individuals. This research suggests that current popular perceptions of autism are conflated with male rather than female stereotypes, and that widely used measures such as the AQ are presenting items meant to measure autistic traits that are simultaneously congruent with male rather than female stereotypes. Future research should include items in measures such as the AQ that align with common autistic female characteristics and behaviors, or create a new version of such measures specifically geared towards females. Finally, while this study identified a significant pattern of male versus female bias towards ASC traits, it did not elucidate why this pattern exists, though several hypotheses were suggested. Understanding why general perceptions of autism are skewed towards males rather than females and educating individuals on female autism presentation is paramount to improve the identification and widespread acceptance of autistic females on the broader community.

# **References**

Adenzato, M., Brambilla, M., Manenti, R., De Lucia, L., Trojano, L., Garofalo, S., Enrici, I., & Cotelli, M. (2017). Gender differences in cognitive Theory of Mind revealed by transcranial direct current stimulation on medial prefrontal cortex. *Scientific Reports, 7*(1), 41219. https://doi.org/10.1038/srep41219

American Psychiatric Association. (2000). *Diagnostic and statistical manual of mental disorders* (4^th^ Eds). American Psychiatric Association

Amodio, D. M. (2014). The neuroscience of prejudice and stereotyping. *Nature Reviews Neuroscience, 15*(10), 670-682. <https://doi.org/10.1038/nrn3800>

Attwood, T. (2006). *The complete guide to Asperger's syndrome.* Jessica Kingsley Publishers.

Bargiela, S., Steward, R., & Mandy, W. (2016). The experiences of late-diagnosed women with autism spectrum conditions: An investigation of the female autism phenotype. *Journal of autism and developmental disorders, 46*(10), 3281-3294. <https://doi.org/10.1007/s10803-016-2872-8>

Baron-Cohen, S. (2002). The extreme male brain theory of autism. Trends in cognitive sciences, 6(6), 248-254. <https://doi.org/10.1016/S1364-6613(02)01904-6>

Baron-Cohen, S., Bowen, D. C., Holt, R. J., Allison, C., Auyeung, B., Lombardo, M. V., Smith, P., & Lai, M.-C. (2015). The “reading the mind in the eyes” test: Complete absence of typical sex difference in~ 400 men and women with autism. *PLoS One, 10*(8), e0136521.

Baron-Cohen, S., Leslie, A. M., & Frith, U. (1985). Does the autistic child have a “theory of mind” ? *Cognition, 21*(1), 37-46. https://doi.org/http://dx.doi.org/10.1016/0010-0277(85)90022-8

Baron-Cohen, S., Wheelwright, S., Skinner, R., Martin, J., & Clubley, E. (2001). The autism-spectrum quotient (AQ): Evidence from Asperger syndrome/high-functioning autism, males and females, scientists and mathematicians. *Journal of autism and developmental disorders, 31*(1), 5-17. <https://doi.org/10.1023/A:1005653411471>

Briton, N. J., & Hall, J. A. (1995). Beliefs about female and male nonverbal communication. *Sex Roles, 32*(1-2), 79-90. <https://doi.org/10.1007/BF01544758>

Cage, E., & Burton, H. (2019). Gender differences in the first impressions of autistic adults. *Autism Research, 12*(10), 1495-1504. <https://doi.org/10.1002/aur.2191>

Cage, E., Di Monaco, J., & Newell, V. (2018). Experiences of autism acceptance and mental health in autistic adults. *Journal of autism and developmental disorders, 48*(2), 473-484. <https://doi.org/10.1007/s10803-017-3342-7>

Cassidy, S. A., Gould, K., Townsend, E., Pelton, M., Robertson, A. E., & Rodgers, J. (2020). Is camouflaging autistic traits associated with suicidal thoughts and behaviours? Expanding the interpersonal psychological theory of suicide in an undergraduate student sample. *Journal of autism and developmental disorders, 50*(10), 3638-3648. <https://doi.org/10.1007/s10803-019-04323-3>

Chevallier, C., Kohls, G., Troiani, V., Brodkin, E. S., & Schultz, R. T. (2012). The social motivation theory of autism. *Trends in Cognitive Sciences, 16*(4), 231-239. https://doi.org/http://dx.doi.org/10.1016/j.tics.2012.02.007

Coleman-Smith, R. S., Smith, R., Milne, E., & Thompson, A. R. (2020). ‘Conflict versus congruence’: A qualitative study exploring the experience of gender dysphoria for adults with autism spectrum disorder. *Journal of autism and developmental disorders, 50*(8), 2643. <https://doi.org/10.1007/s10803-019-04296-3>

Dean, M., Harwood, R., & Kasari, C. (2017). The art of camouflage: Gender differences in the social behaviors of girls and boys with autism spectrum disorder. *Autism, 21*(6), 678-689. <https://doi.org/10.1177/1362361316671845>

De Goede, I. H., Branje, S. J., & Meeus, W. H. (2009). Developmental changes in adolescents’ perceptions of relationships with their parents. *Journal of youth and adolescence, 38*(1), 75-88. <https://10.1007/s10964-008-9286-7>

Devine, R. T., & Hughes, C. (2013). Silent films and strange stories: Theory of mind, gender, and social experiences in middle childhood. *Child Development, 84*(3), 989-1003.

Dickter, C. L., Burk, J. A., Zeman, J. L., & Taylor, S. C. (2020). Implicit and explicit attitudes toward autistic adults. *Autism in Adulthood*, *2*(2), 144-151. <https://doi.org/10.1089/aut.2019.0023>

Duvekot, J., van der Ende, J., Verhulst, F. C., Slappendel, G., van Daalen, E., Maras, A., & Greaves-Lord, K. (2017). Factors influencing the probability of a diagnosis of autism spectrum disorder in girls versus boys. *Autism, 21*(6), 646-658. <https://doi.org/10.1177/1362361316672178>

Dworzynski, K., Ronald, A., Bolton, P., & Happé, F. (2012). How different are girls and boys above and below the diagnostic threshold for autism spectrum disorders?. *Journal of the American Academy of Child & Adolescent Psychiatry, 51*(8), 788-797. <https://doi.org/10.1016/j.jaac.2012.05.018>

Eagly, A. H., & Wood, W. (2013). The nature–nurture debates: 25 years of challenges in understanding the psychology of gender. *Perspectives on Psychological Science, 8*(3), 340-357. [https://doi.org/10.1177/1745691613484767](https://doi.org/10.1177%2F1745691613484767)

Ellemers, N. (2018). Gender stereotypes. *Annual review of psychology, 69*, 275-298. [https://doi.org/10.1146/annurev- psych- 122216- 011719](https://doi.org/10.1146/annurev-%20psych-%20122216-%20011719)

Frazier, T. W., Georgiades, S., Bishop, S. L., & Hardan, A. Y. (2014). Behavioral and cognitive characteristics of females and males with autism in the Simons Simplex Collection. *Journal of the American Academy of Child & Adolescent Psychiatry, 53*(3), 329-340. <https://doi.org/10.1016/j.jaac.2013.12.004>

Garcia-Retamero, R., & Lopez-Zafra, E. (2009). Causal attributions about feminine and leadership roles: A cross-cultural comparison. *Journal of Cross-Cultural Psychology, 40*(3), 492-509. <https://doi.org/10.1177/0022022108330991>

Garner, A., Jones, S., & Harwood, V. (2015). Authentic representations or stereotyped ‘outliers’: using the CARS2 to assess film portrayals of Autism Spectrum Disorders. *International Journal of Culture and Mental Health, 8*(4), 414-425. <https://doi.org/10.1080/17542863.2015.1041993>

Geelhand, P., Bernard, P., Klein, O., Van Tiel, B., & Kissine, M. (2019). The role of gender in the perception of autism symptom severity and future behavioural development. *Molecular autism, 10*(1), 16. <https://doi.org/10.1186/s13229-019-0266-4>

Giarelli, E., Wiggins, L. D., Rice, C. E., Levy, S. E., Kirby, R. S., & Pinto-Martin, J., (2010). Sex differences in the evaluation and diagnosis of autism spectrum disorders among children. *Disability and Health Journal,* *3*(2), 107–116. <https://doi.org/10.1016/j.dhjo.2009.07.001>

Gillespie-Lynch, K., Hotez, E., Zajic, M., Riccio, A., DeNigris, D., Kofner, B., ... & Luca, K. (2020). Comparing the writing skills of autistic and non-autistic university students: A collaboration with autistic university students. *Autism, 24*(7), 1898-1912. <https://doi.org/10.1177/1362361320929453>

Greenberg, D. M., Warrier, V., Allison, C., & Baron-Cohen, S. (2018). Testing the Empathizing–Systemizing theory of sex differences and the Extreme Male Brain theory of autism in half a million people. *Proceedings of the National Academy of Sciences, 115*(48), 12152-12157. <https://doi.org/10.1073/pnas.1811032115>

Greenwald, A. G., McGhee, D. E., & Schwartz, J. L. (1998). Measuring individual differences in implicit cognition: the implicit association test. *Journal of personality and social psychology, 74*(6), 1464

Greenwald, A. G., Poehlman, T. A., Uhlmann, E. L., & Banaji, M. R. (2009). Understanding and using the Implicit Association Test: III. Meta-analysis of predictive validity. *Journal of personality and social psychology, 97*(1), 17. <https://doi.org/10.1037/a0015575>

Heilman, M. E., & Chen, J. J. (2005). Same behavior, different consequences: reactions to men's and women's altruistic citizenship behavior. *Journal of Applied Psychology, 90*(3), 431. <https://doi.org/10.1037/0021-9010.90.3.431>

Hess, U., Senécal, S., Kirouac, G., Herrera, P., Philippot, P., & Kleck, R. E. (2000). Emotional expressivity in men and women: Stereotypes and self-perceptions. *Cognition & Emotion, 14*(5), 609-642. <https://doi.org/10.1080/02699930050117648>

Hiller, R. M., Young, R. L., & Weber, N. (2014). Sex differences in autism spectrum disorder based on DSM-5 criteria: evidence from clinician and teacher reporting. *Journal of abnormal child psychology, 42*(8), 1381-1393. <https://doi.org/10.1007/s10802-014-9881-x>

Hull, L., Petrides, K. V., & Mandy, W. (2020). The female autism phenotype and camouflaging: A narrative review. *Review Journal of Autism and Developmental Disorders, 7,* 1-12. <https://doi.org/10.1007/s40489-020-00197-9>

Jackson, E. F., & Bussey, K. (2020). Under Pressure: Differentiating Adolescents’ Expectations Regarding Stereotypic Masculine and Feminine Behavior. *Sex Roles*, 83(6), 303-314. <https://doi.org/10.1007/s11199-019-01113-0>

Jones, S. C., Gordon, C. S., Akram, M., Murphy, N., & Sharkie, F. (2021). Inclusion, Exclusion and Isolation of Autistic People: Community Attitudes and Autistic People’s Experiences. *Journal of Autism and Developmental Disorders*, 1-12. <https://doi.org/10.1007/s10803-021-04998-7>

Kanner, L. (1943). Autistic disturbances of affective contact. *Nervous Child, 2,* 217-250.

Krahn, T. M., & Fenton, A. (2012). The extreme male brain theory of autism and the potential adverse effects for boys and girls with autism. *Journal of bioethical inquiry, 9*(1), 93-103. <https://doi.org/10.1007/s11673-011-9350-y>

Kenyon, S. (2014). Autism in pink: Qualitative research report. <http://www.www.naspink2.webeden.co.uk>. Accessed April 10, 2021.

Kim, D. Y. (2003). Voluntary controllability of the implicit association test (IAT). *Social Psychology Quarterly*, *66*(1) 83-96. <https://doi.org/10.2307/3090143>

Kirkovski, M., Enticott, P. G., & Fitzgerald, P. B. (2013). A review of the role of female gender in autism spectrum disorders. *Journal of autism and developmental disorders, 43*(11), 2584-2603.

Kite, M. E., Deaux, K., & Haines, E. L. (2008). *Gender stereotypes*. Praeger Publishers/Greenwood Publishing Group.

Kopp, S., & Gillberg, C. (2011). The Autism Spectrum Screening Questionnaire (ASSQ)-Revised Extended Version (ASSQ-REV): an instrument for better capturing the autism phenotype in girls? A preliminary study involving 191 clinical cases and community controls. *Research in developmental disabilities, 32*(6), 2875-2888. <https://doi.org/10.1016/j.ridd.2011.05.017>

Krahn, T. M., & Fenton, A. (2012). The extreme male brain theory of autism and the potential adverse effects for boys and girls with autism. *Journal of bioethical inquiry, 9*(1), 93-103. <https://doi.org/10.1007/s11673-011-9350-y>

Lai, M. C., Lombardo, M. V., Auyeung, B., Chakrabarti, B., & Baron-Cohen, S. (2015). Sex/gender differences and autism: setting the scene for future research. *Journal of the American Academy of Child & Adolescent Psychiatry, 54*(1), 11-24. <https://doi.org/10.1016/j.jaac.2014.10.003>

Lane, K. A., Banaji, M. R., Nosek, B. A., & Greenwald, A. G. (2007). *Understanding and Using the Implicit Association Test: IV: What We Know (So Far) about the Method.* The Guilford Press.

Lehnhardt, F. G., Falter, C. M., Gawronski, A., Pfeiffer, K., Tepest, R., Franklin, J., & Vogeley, K. (2016). Sex-related cognitive profile in autism spectrum disorders diagnosed late in life: implications for the female autistic phenotype. *Journal of Autism and Developmental Disorders, 46*(1), 139-154. <https://10.1007/s10803-015-2558-7>

Loomes, R., Hull, L., & Mandy, W. P. L. (2017). What is the male-to-female ratio in autism spectrum disorder? A systematic review and meta-analysis. *Journal of the American Academy of Child & Adolescent Psychiatry*, *56*(6), 466-474. <https://doi.org/10.1016/j.jaac.2017.03.013>

Losh, M., & Capps, L. (2006). Understanding of emotional experience in autism: Insights from the personal accounts of high-functioning children with autism. *Developmental Psychology, 42*(5), 809–818. <https://doi.org/10.1037/0012-1649.42.5.809>

Luengo Kanacri, B. P., Pastorelli, C., Eisenberg, N., Zuffianò, A., & Caprara, G. V. (2013). The development of prosociality from adolescence to early adulthood: The role of effortful control. *Journal of personality, 81*(3), 302-312. <https://doi.org/10.1111/jopy.12001>

Mandy, W., & Lai, M. C. (2017). Towards sex-and gender-informed autism research*. 21*(6), 643-645. <https://doi.org/10.1177/1362361317706904>

Mazurek, M. O., Curran, A., Burnette, C., & Sohl, K. (2019). ECHO Autism STAT: accelerating early access to autism diagnosis. *Journal of autism and developmental disorders, 49*(1), 127-137.

Milner, V., McIntosh, H., Colvert, E., & Happé, F. (2019). A qualitative exploration of the female experience of autism spectrum disorder (ASD). *Journal of autism and developmental disorders, 49*(6), 2389-2402. <https://doi.org/10.1007/s10803-019-03906-4>

Milton, D. E. (2012). On the ontological status of autism: the ‘double empathy problem’. *Disability & Society, 27*(6), 883-887. <https://doi.org/10.1080/09687599.2012.710008>

Nordahl-Hansen, A., Øien, R. A., & Fletcher-Watson, S. (2018). Pros and cons of character portrayals of autism on TV and film. *Journal of Autism and Developmental Disorders, 48*(2), 635-636. <https://doi.org/10.1007/s10803-017-3390-z>

Nosek, B. A., Banaji, M. R., & Greenwald, A. G. (2002). Harvesting implicit group attitudes and beliefs from a demonstration web site. *Group Dynamics: Theory, Research, and Practice, 6*(1), 101. <https://doi.org/10.1037/1089-2699.6.1.101>

Pascual-Miguel, F. J., Agudo-Peregrina, Á. F., & Chaparro-Peláez, J. (2015). Influences of gender and product type on online purchasing. *Journal of Business Research, 68*(7), 1550-1556. <https://doi.org/10.1016/j.jbusres.2015.01.050>

Park, H. R., Lee, J. M., Moon, H. E., Lee, D. S., Kim, B. N., Kim, J., & Paek, S. H. (2016). A short review on the current understanding of autism spectrum disorders. *Experimental neurobiology*, *25*(1), 1-13. <https://doi.org/10.5607/en.2016.25.1.1>

Parkins, R. (2012). *Gender and emotional expressiveness: An analysis of prosodic features in emotional expression.* Griffith University.

Pellicano, E., Dinsmore, A., & Charman, T. (2014). What should autism research focus upon? Community views and priorities from the United Kingdom. *Autism, 18*(7), 756-770. <https://doi.org/10.1177/1362361314529627>

Ridley, R. (2019). Some difficulties behind the concept of the ‘Extreme male brain in autism research. A theoretical review. *Research in Autism Spectrum Disorders, 57*, 19-27. <https://doi.org/10.1016/j.rasd.2018.09.007>

Ruzich, E., Allison, C., Smith, P., Watson, P., Auyeung, B., Ring, H., Baron-Cohen, S. (2015). Measuring autistic traits in the general population: A systematic review of the Autism-Spectrum Quotient (AQ) in a non-clinical population sample of 6,900 typical adult males and females. *Molecular Autism, 6*(1), 1. <https://doi.org/10.1186/2040-2392-6-2>

Rynkiewicz, A., Schuller, B., Marchi, E., Piana, S., Camurri, A., Lassalle, A., & Baron-Cohen, S. (2016). An investigation of the ‘female camouflage effect in autism using a computerized ADOS-2 and a test of sex/gender differences. *Molecular autism, 7*(1), 10. <https://doi.org/10.1186/s13229-016-0073-0>

Sutherland, R., Hodge, A., Bruck, S., Costley, D., & Klieve, H. (2017). Parent-reported differences between school-aged girls and boys on the autism spectrum. *Autism, 21*(6), 785-794. <https://doi.org/10.1177/1362361316668653>

Top 1000 popular babynames in 2000 - BabyNames.it - 100% American baby name. Babynames.it. (2021). Retrieved 18 May 2021, from <http://www.babynames.it/top100/2/year-2000.html>.

Watson, L. E. (2014). 'Living life in the moment': Chronic stress and coping among families of high-functioning adolescent girls with autism spectrum disorder. ProQuest Information & Learning). *Dissertation Abstracts International Section A: Humanities and Social Sciences, 76*.

White, D., Hillier, A., Frye, A., & Makrez, E. (2019). College students’ knowledge and attitudes towards students on the autism spectrum. *Journal of Autism and Developmental Disorders, 49*(7), 2699-2705. <https://doi.org/10.1007/s10803-016-2818-1>

Whitlock, A., Fulton, K., Lai, M. C., Pellicano, E., & Mandy, W. (2020). Recognition of girls on the autism spectrum by primary school educators: an experimental study. *Autism Research, 13*(8), 1358-1372. <https://doi.org/10.1002/aur.2316>

Willey, L. H. (2014). *Pretending to be normal: Living with asperger's syndrome (autism spectrum disorder) expanded edition.* Jessica Kingsley Publishers.

Yaull-Smith, D. (2008). Girls on the spectrum. *Communication. National Autistic Society*.
