# Supplementary material for: Autism, thy name is man: Exploring implicit and explicit gender bias in autism perceptions
Source: PLoS One. 2023 Aug 23;18(8):e0284013. doi: 10.1371/journal.pone.0284013 (PMC10446214; doi:10.1371/journal.pone.0284013)
Supplement: Attachment — Submitted filename: Reviewer replies.docx [file pone.0284013.s002.docx]

Dear Asem, Mathew and Sabina.

We would like to thank all of you taking the time to review this work and your very constructive, helpful and thoughtful comments. We have responded to each of your points below (in red) and made changes to the MS in the appropriate places. We are confident this work is much stronger for your input and would gladly welcome any further feedback. We would also like to take the opportunity to inform then editor, that we have in line with R2’s suggestion for additional stats check by a statistician, done this, therefore there has now been an additional author added to the paper. Additionally data has now also been added to the OSF and link included in the MS.

Many thanks , the authors.


Reviewer #1

The recent publications most relevant to this argument, those introducing the unbiased Comprehensive Autistic Trait Inventory (English & al., 2021, doi: 10.1186/s13229-021-00445-7) and detailing the differences in categorical diagnoses between girls and boys with similar genetic loadings for autism (Burrows & al., 2022, doi: 10.1016/j.biopsych.2022.05.027), are not mentioned.

Thank you for directing us to these very relevant works that we missed. We have now included these works in the MS; reference to Burrows & al., 2022 can be found on page 23, and English & al., 2021 on page 22.

The culture (or cultures) from which survey respondents are drawn is not specified. This detail is important because gender norms vary across cultures.

This is a very good point, which we had not considered. Our participants were mostly (64%) form the UK, 9% American, with the remaining 27% coming from across various countries, mostly in Europe and Asia. We have now included this information in the Methods, for your own reference we have also included the full break down for you below.

In the Implicit Association Test, an error penalty of 600 ms seems arbitrary and liable to skew averages, and both this penalty and the replacement with the current block's mean reaction time seem liable to skew means and variances. Why not just omit error trials from the computation of mean reaction time? In the report of mean reaction times by condition, are the units milliseconds (ms)? Specify. And what is the proper number of significant figures; can the hardware actually measure intervals as short as tenths of microseconds? Typical USB keyboard scan intervals are on the order of a millisecond.

Yes, reponses are given in ms. We noticed an error in that the reported means were the totals of the 2 means form the relevant blocks, rather than ture means, were reported so these have been replaced. Inferentials remain unaffected. The 600 ms penalty is not arbitrary, it is the recommended procedure for an IAT as per Greenwald et al., 2009; Lane et al., 2007; Nosek et al., 2002. Error trials should not be omitted as one is interested in both RT and accuracy. Adding a 600ms penalty to incorrect scores, is a well-established formula, Greenwald et al., 2009,for instance has been cited over 9000 times in total. Google scholar suggest there are upwards of 400,000 research papers that use or discuss the IAT.


The text reports "One AQ item was identified as differing significantly across the conditions at the .05 alpha level, and a further 3 at the .01 alpha level" but the tail probabilities in Table 2 do not conform to this description, and the data for AQ item #39 in particular seem not to argue at all strongly again (p[f f reviews) not the null hypothesis. Also in Table 2, it's unclear why the Bayes factor is missing in the first (AQ item #1) data row, the narrow column widths cause data to be broken across rows, and it might be simpler to report a single Bayes factor than to report a pair of reciprocals.

Apologies, there was a typo. One item differed below the .05 alpha level, and a further 3 were identified as marginal (between .05 – .10) Bayes factors have now been removed entirely although not included we did run further Mann-Whitney U tests (which could be added) which show 3 of the 4 items in question differ below the .05 level, while one (item 34 came out marginal at .055), though we chose not to include this as this was not pre-registered, and we didn’t want to double dip reporting 2 lots of analyses on the same variables.

Statements of interpretation such as "participants found it easier to associate autistic words with male names" and "These empirical findings reveal a tendency in the average person to associate autistic traits with males compared to females" should be confined to the discussion section.

We have amended these as requested.

The manuscript file asserts "The data that support the findings of this study are available on request from the corresponding author. The data are not publicly available due to privacy or ethical restrictions." Yet the "Data Availability" question is answered "Yes - all data are fully available without restriction". This contradictory pair of responses violates the instruction "Important: Stating `data available on request from the author' is not sufficient. If your data are only available upon request, select `No' for the first question and explain your exceptional situation in the text box."

Apologies for this. There were crossed wires between the co-authors, the data is available on the OSF, and the link is in the paper.

I have annotated the authors' manuscript with suggested (tracked) changes and marginal notes for the authors' use in revision and am uploading this anonymously annotated manuscript as part of this review; please see this annotated file is forwarded to the authors.

We are very grateful for you taking the time to do this. It is very much appreciated. All changes are incorporated.


Reviewer #2: WHAT IS THE STUDY ABOUT? Female stereotyped behaviour stands in stark contrast to typically recognised autistic traits. Women display more emotional expression and communication in social situations, thereby masking diagnosis and delaying intervention. The abstract and Introduction handle the above summary. Abbreviations should include the expanded form.

METHOD: Population-based study appropriate. Sample size of 300 was selected by appropriate power analysis. Cross-cultural, online and social media searches however needed further elaboration on the methodology of collecting data. Age range mentioned. Ethical approval taken. Informed consent was taken. There was no clarification about the participations previous knowledge of Autism which could influence the results.

RESULTS: Statistical Analysis not checked: needs review by a statistician.
Results support conclusions. Limitations have been discussed. The study is on a valid topic and very essential for general public understanding to remove stereotyped assumptions. Important also for health sector, education and researchers. This study shows the need for early diagnosis and will help to reduce the demand on females with autism for higher level of functional needs in behaviour, development, thus affecting mental health. The author has also mentioned objection about the Male brain term.

CONCLUSION: Data is enough to recreate the analysis of the selected points by the author. Previous knowledge on autism is necessary to give a correct picture.
RECOMMENDED CORRECTIONS: "Greater communality'' to be replaced by "greater communication ability
Needs correction "Non autistic characteristic are talkative". High functioning verbal women are also talkative in their own interest area. This criterion needs modification.
Abbreviations need to be given in expanded form at the initial stage.
PLAGIARISM: Not checked


Thank you for your helpful comments, we have addressed all of these in the MS, abbreviations are now expanded on, as are our sampling procedure.

Regarding participants knowledge of autism this is an excellent point. Its something we did actually collect data on originally but never delved in to too much in the paper, this has now been described adequately in the methods (detail given for you below)and results, where we control for this with in further IAT anyalsis. We thank the reviewer for this observation.

We asked participants to indicate how much knowledge they had of autism using a 5-point Likert scale (1 – Not at all; 2 – A little; 3 – A moderate amount; 4 – A lot; 5 – A great deal). We have now added this information in the method section.

Thank you also for spotting those typos and other errors we missed. They have all been concerted.
