# Supplementary material for: Autism, thy name is man: Exploring implicit and explicit gender bias in autism perceptions
Source: PLoS One. 2023 Aug 23;18(8):e0284013. doi: 10.1371/journal.pone.0284013 (PMC10446214; doi:10.1371/journal.pone.0284013)
Supplement: Attachment — Submitted filename: Reviewer comments.docx [file pone.0284013.s003.docx]

==============================
Authors have carefully revised the manuscript and is appreciated. I would like to add an additional comment to the authors which will be benefited to the readers. There are many reports from the genetic studies, that indicated overrepresentation of autism in males compare to females are likely due to genetic factors. Subsequently, if there is sex linked genes, logically, overrepresentation of autism in males makes sense. I would appreciate if authors could briefly address this point as well. 
==============================

Thank you very much for this comment. You are absolutely right, this is indeed an important area of research that may indeed inform public opinion. I have included two additional prominent theories of why there may be a female protective factor with regards to genetic liability for autism, and why males may be more likely to develop autism using theories relating to genetic differences sex wise in neural plasiticity. I have also included research that suggests that if females are diagnosed younger and using standardised assessments the sex ratio is less pronounced and females have no differences in cognitive functioning compared to males. This suggests that there is a complex relationship between gender, sex and autism that may not be clear with regards to genetic liability. I hope this makes sense and thank you for this comment.
